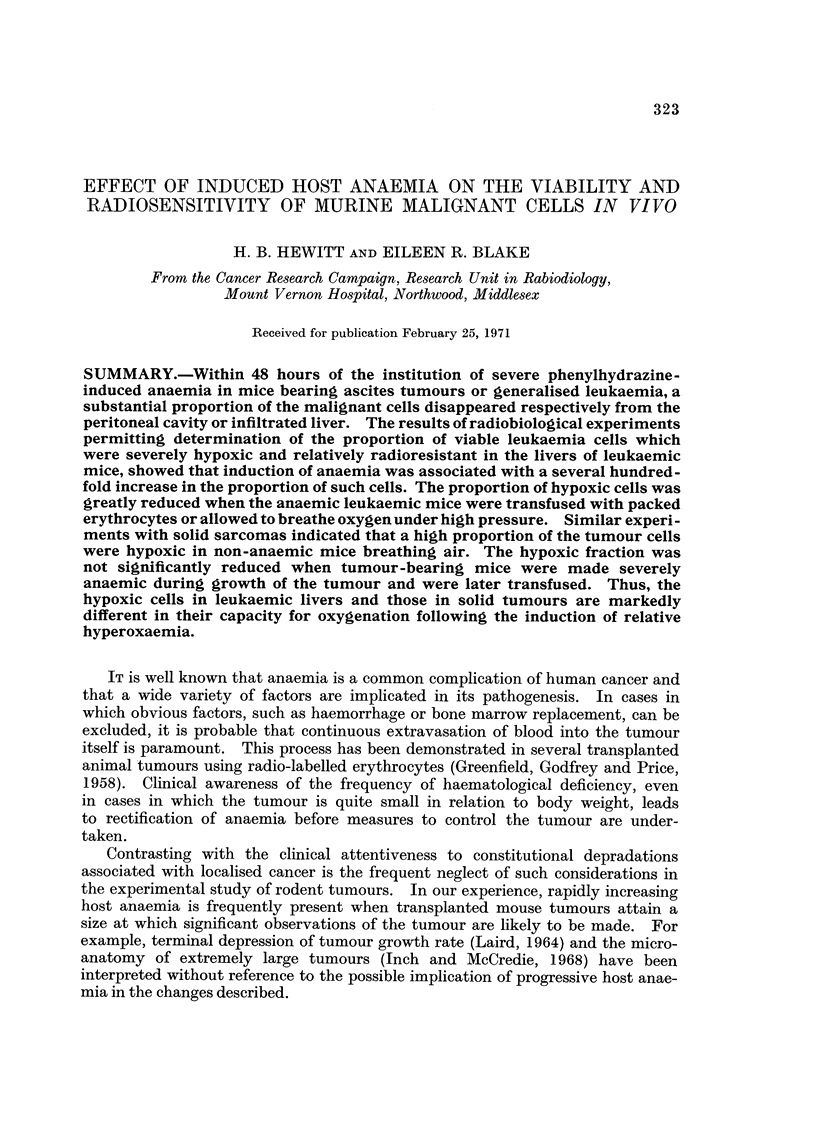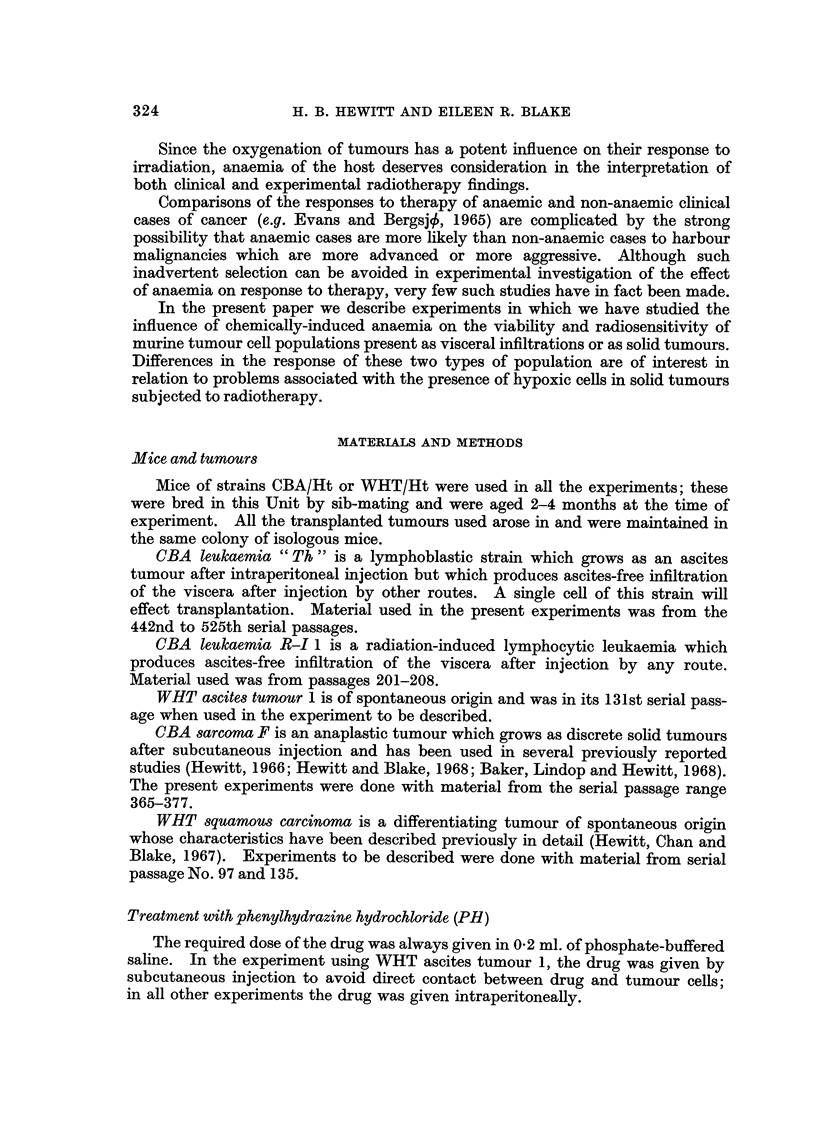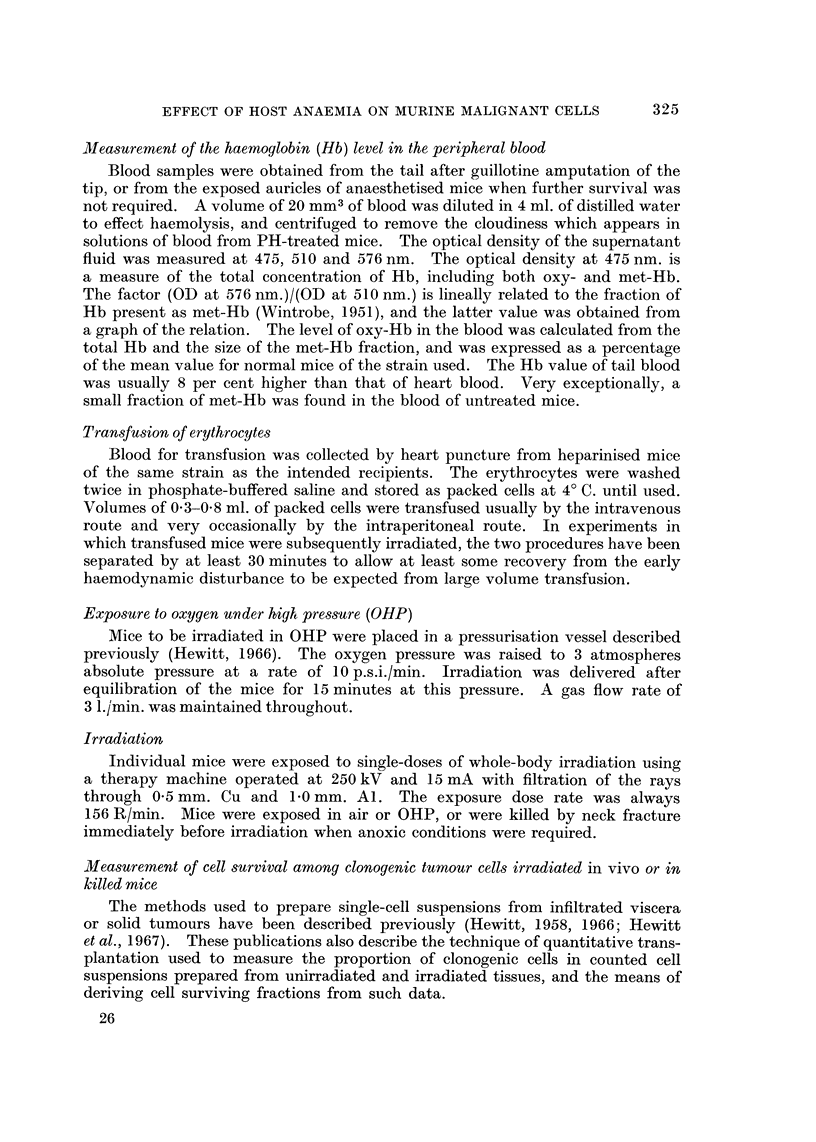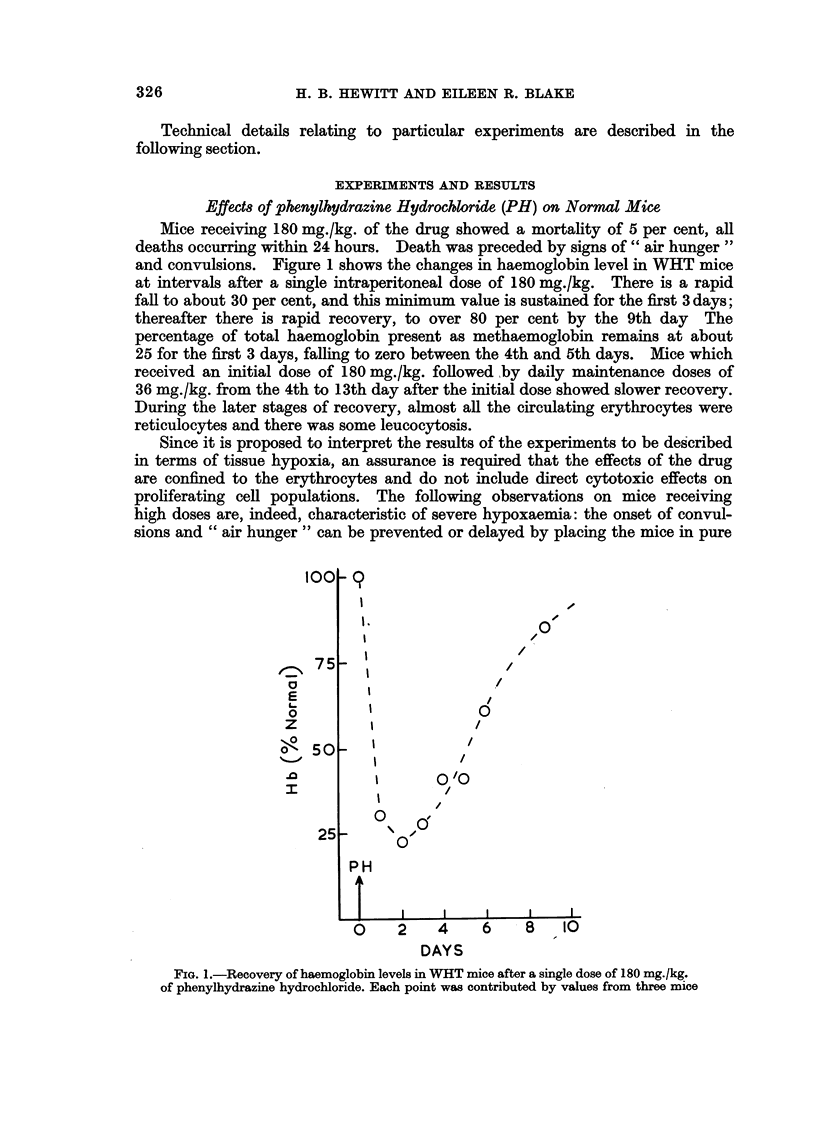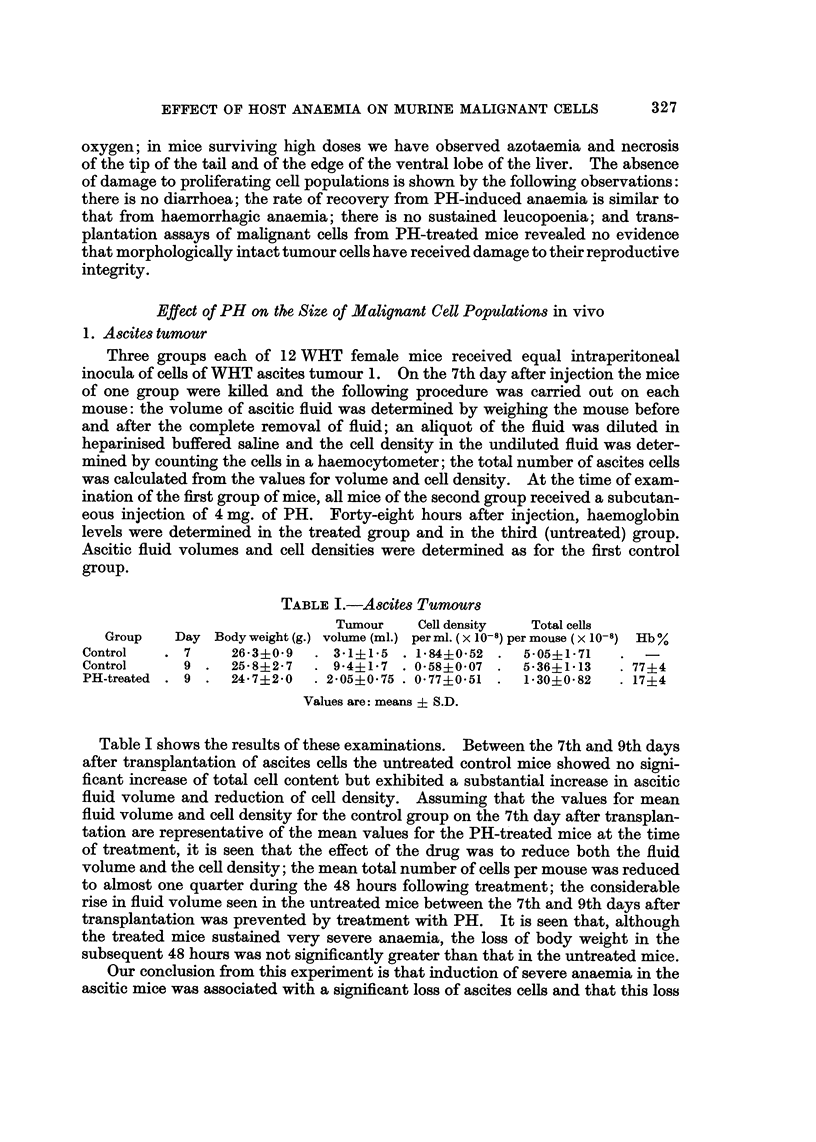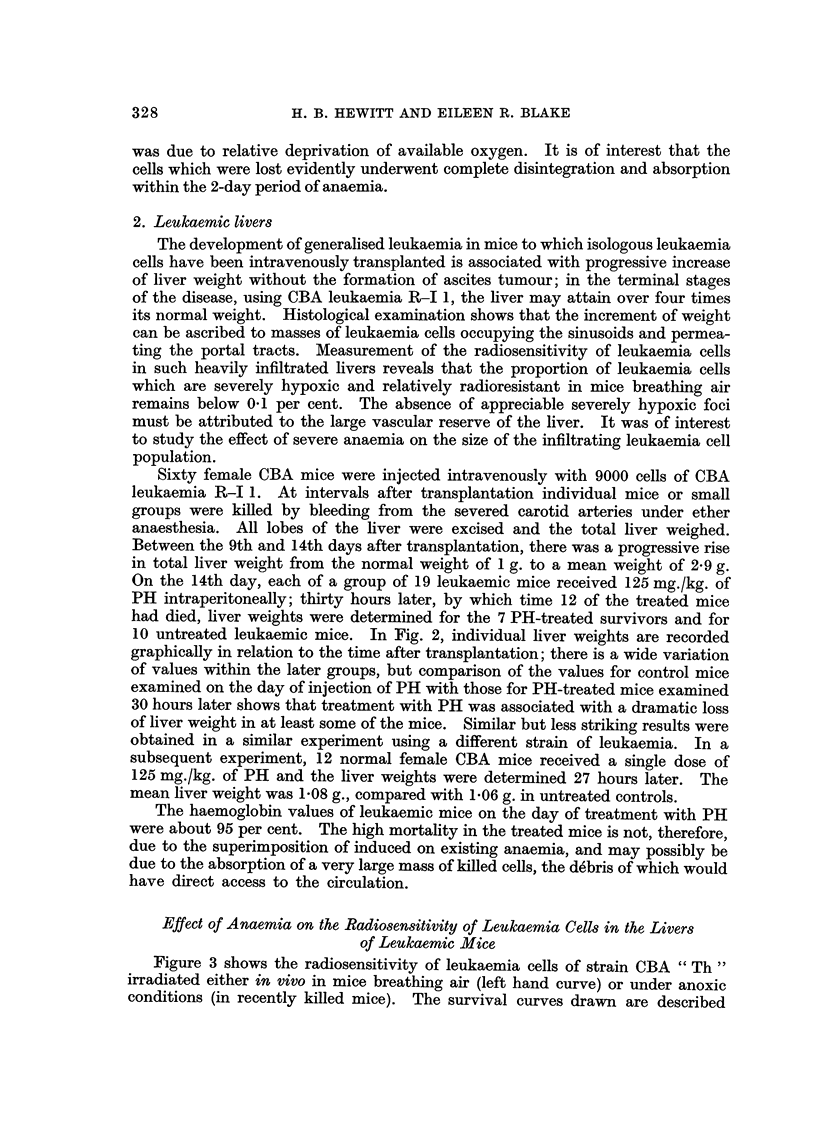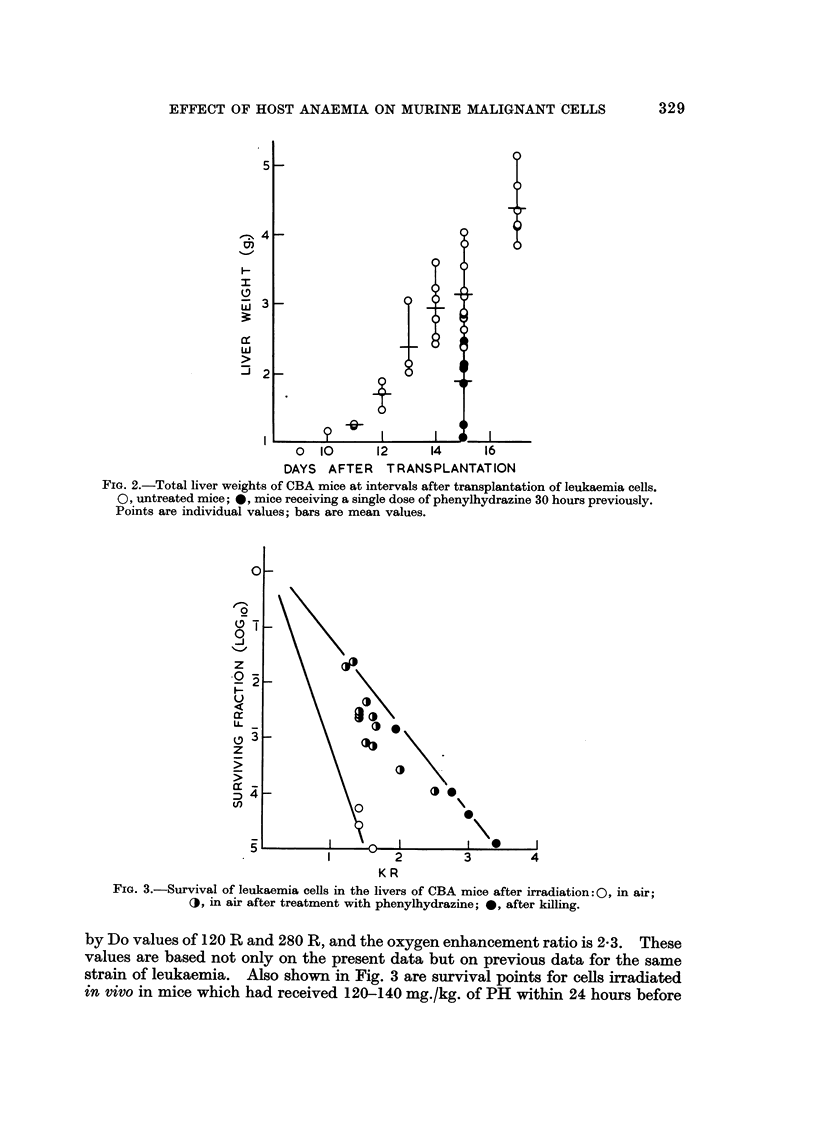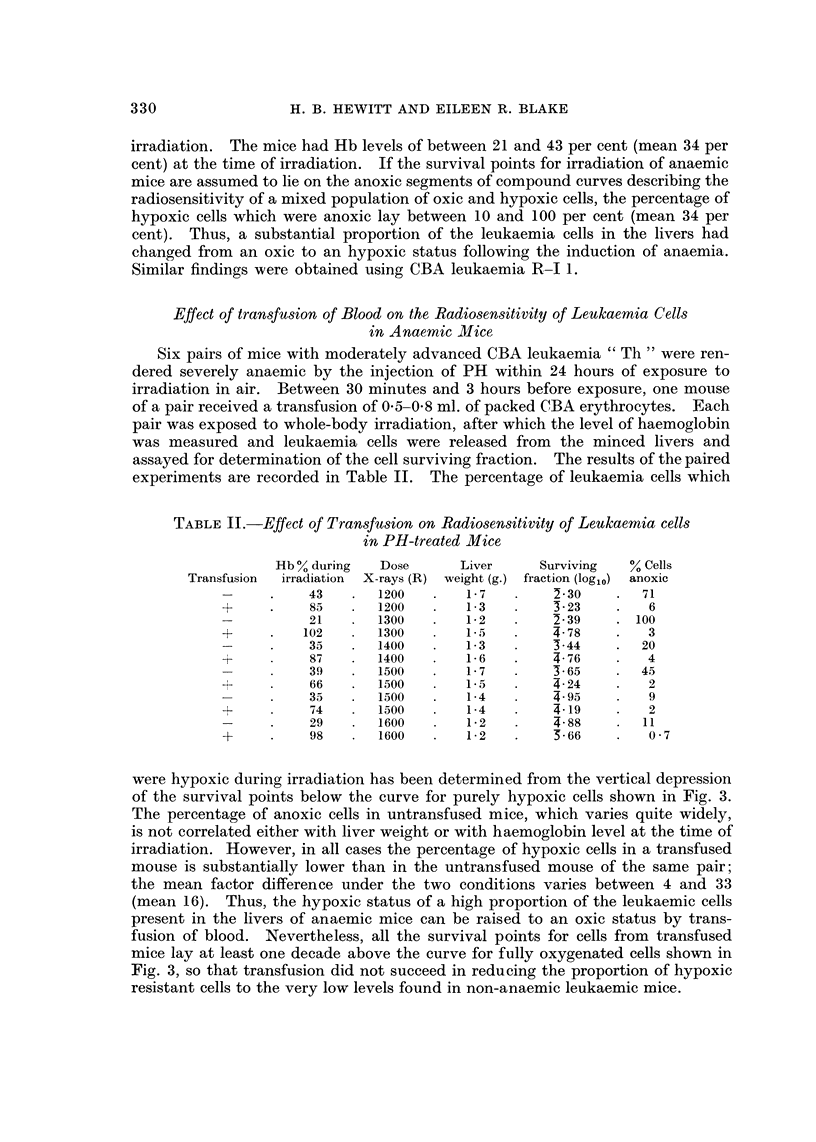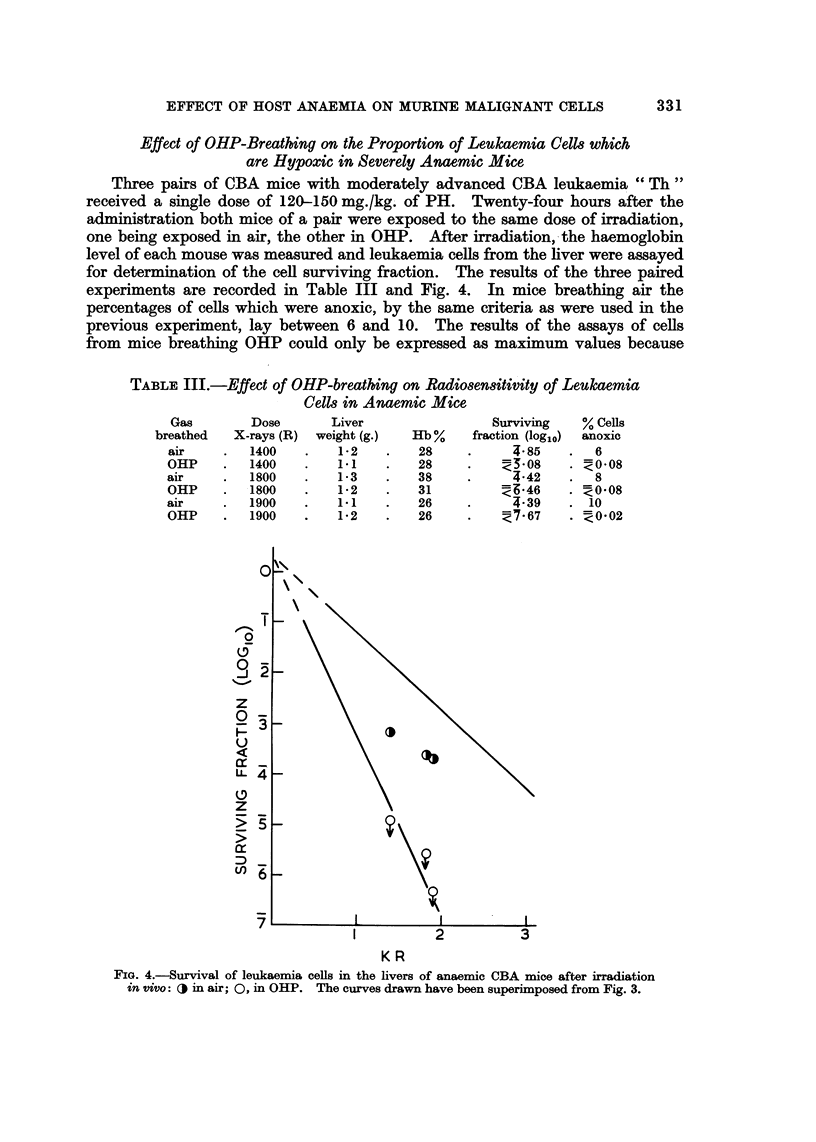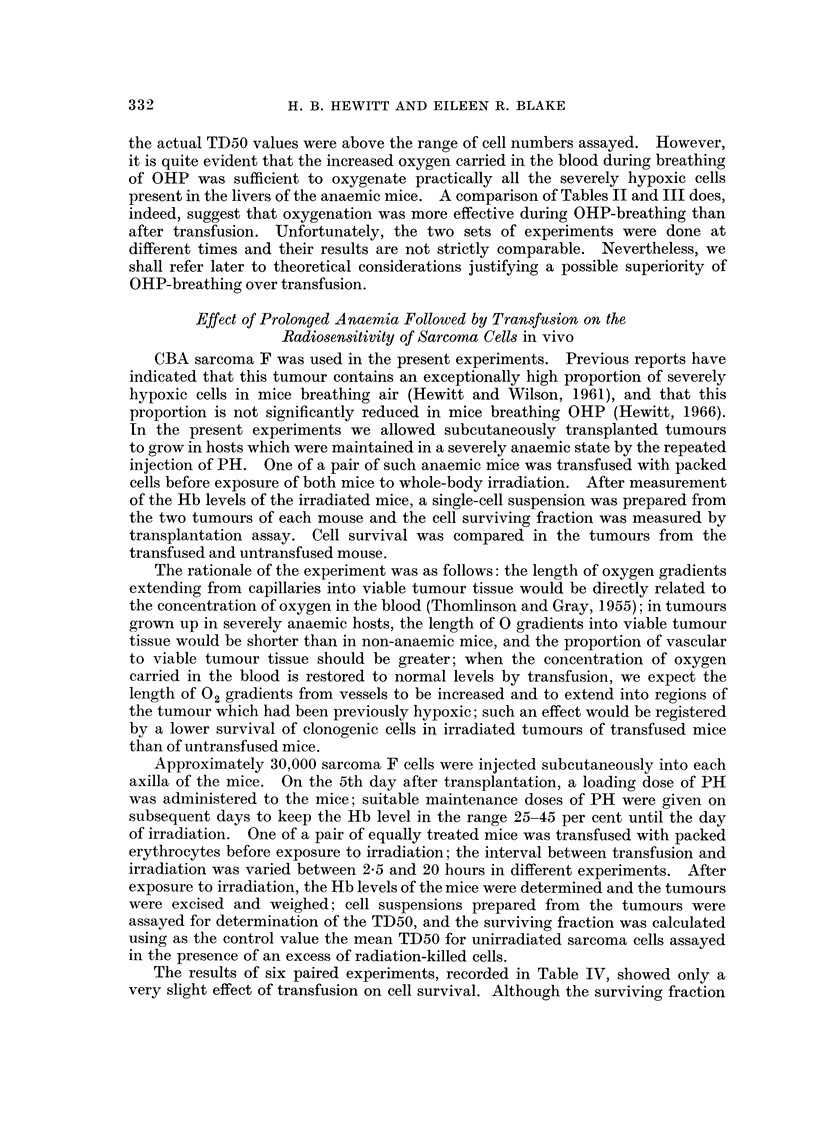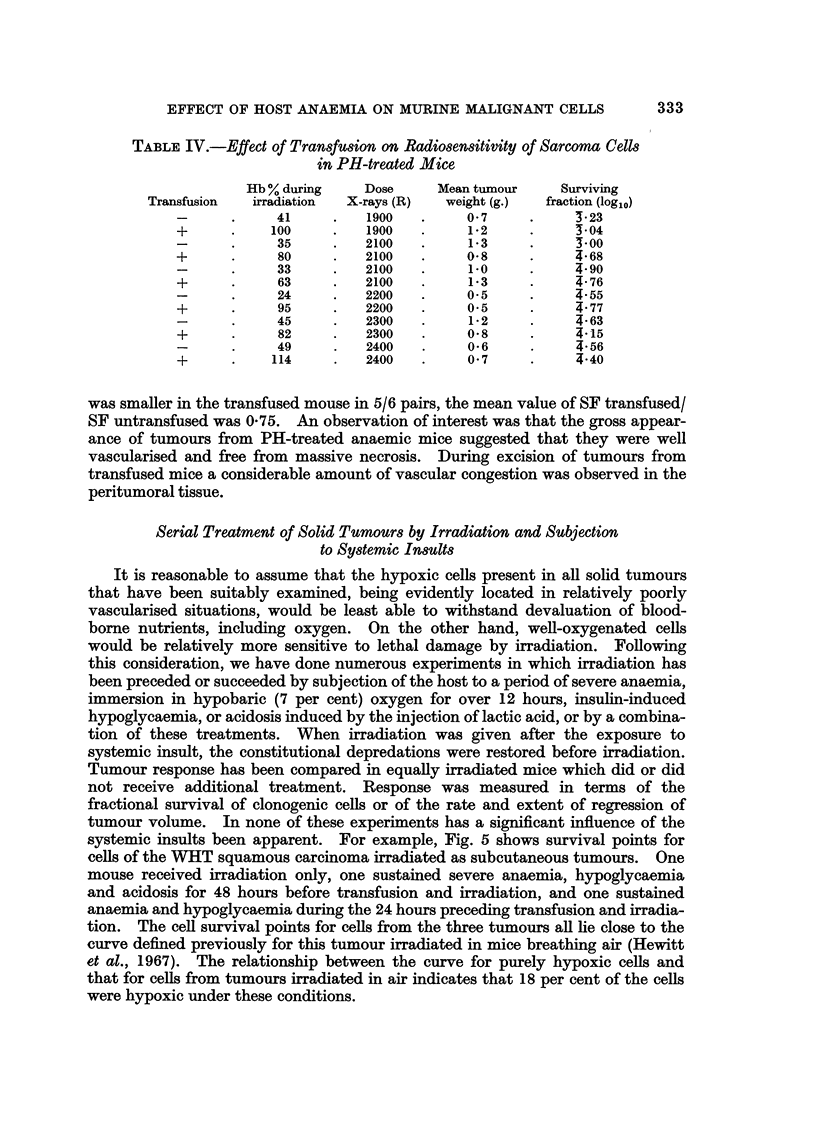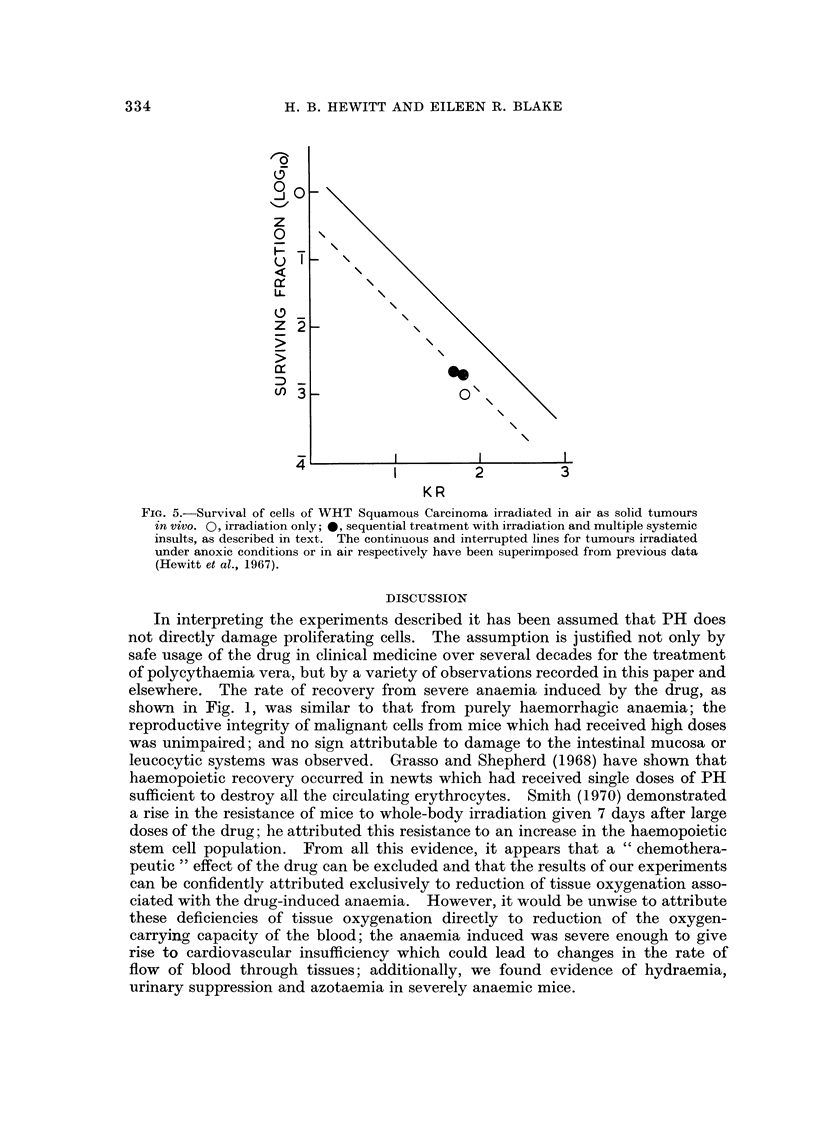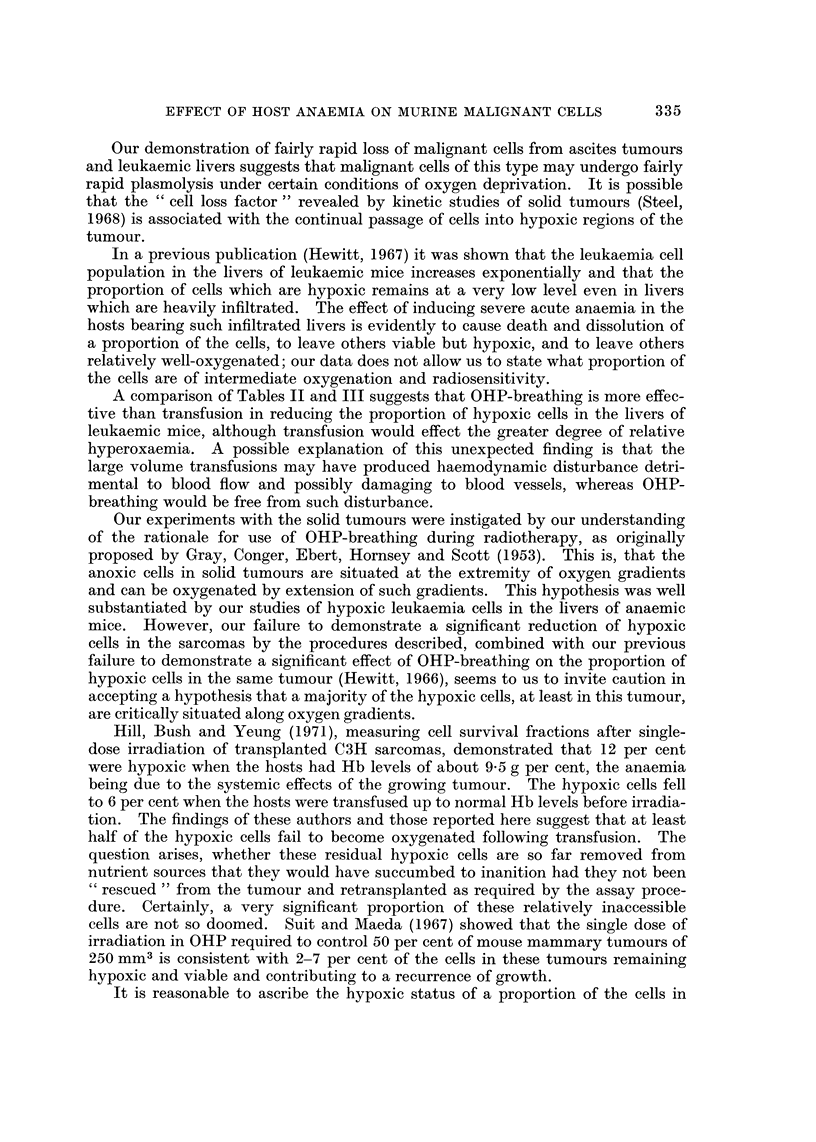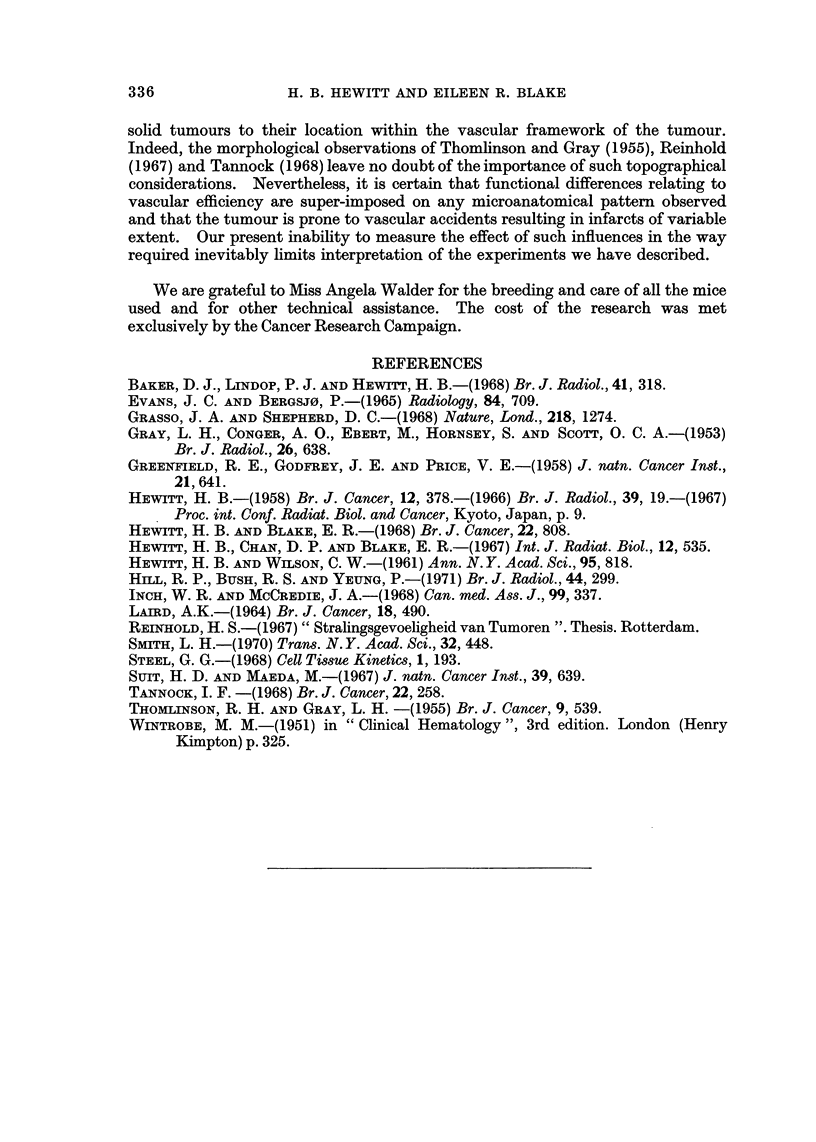# Effect of Induced Host Anaemia on the Viability and Radiosensitivity of Murine Malignant Cells in vivo

**DOI:** 10.1038/bjc.1971.42

**Published:** 1971-06

**Authors:** H. B. Hewitt, Eileen R. Blake

## Abstract

Within 48 hours of the institution of severe phenylhydrazineinduced anaemia in mice bearing ascites tumours or generalised leukaemia, a substantial proportion of the malignant cells disappeared respectively from the peritoneal cavity or infiltrated liver. The results of radiobiological experiments permitting determination of the proportion of viable leukaemia cells which were severely hypoxic and relatively radioresistant in the livers of leukaemic mice, showed that induction of anaemia was associated with a several hundredfold increase in the proportion of such cells. The proportion of hypoxic cells was greatly reduced when the anaemic leukaemic mice were transfused with packed erythrocytes or allowed to breathe oxygen under high pressure. Similar experi - ments with solid sarcomas indicated that a high proportion of the tumour cells were hypoxic in non-anaemic mice breathing air. The hypoxic fraction was not significantly reduced when tumour-bearing mice were made severely anaemic during growth of the tumour and were later transfused. Thus, the hypoxic cells in leukaemic livers and those in solid tumours are markedly different in their capacity for oxygenation following the induction of relative hyperoxaemia.


### EFFECT OF INDUCED HOST ANAEMIA ON THE VIABILITY AND RADIOSENSITIVITY OF MURINE MALIGNANT CELLS *IN VIVO*

H. B. HEWITT AND EILEEN R. BLAKE

*From the Cancer Research Campaign, Research Unit in Rabiodiology, Mount Vernon Hospital, Northwood, Middlesex*

Received for publication February 25, 1971

**SUMMARY.—Within 48 hours of the institution of severe phenylhydrazine-induced anaemia in mice bearing ascites tumours or generalised leukaemia, a substantial proportion of the malignant cells disappeared respectively from the peritoneal cavity or infiltrated liver. The results of radiobiological experiments permitting determination of the proportion of viable leukaemia cells which were severely hypoxic and relatively radioresistant in the livers of leukaemic mice, showed that induction of anaemia was associated with a several hundred-fold increase in the proportion of such cells. The proportion of hypoxic cells was greatly reduced when the anaemic leukaemic mice were transfused with packed erythrocytes or allowed to breathe oxygen under high pressure. Similar experiments with solid sarcomas indicated that a high proportion of the tumour cells were hypoxic in non-anaemic mice breathing air. The hypoxic fraction was not significantly reduced when tumour-bearing mice were made severely anaemic during growth of the tumour and were later transfused. Thus, the hypoxic cells in leukaemic livers and those in solid tumours are markedly different in their capacity for oxygenation following the induction of relative hyperoxaemia.**

IT is well known that anaemia is a common complication of human cancer and that a wide variety of factors are implicated in its pathogenesis. In cases in which obvious factors, such as haemorrhage or bone marrow replacement, can be excluded, it is probable that continuous extravasation of blood into the tumour itself is paramount. This process has been demonstrated in several transplanted animal tumours using radio-labelled erythrocytes (Greenfield, Godfrey and Price, 1958). Clinical awareness of the frequency of haematological deficiency, even in cases in which the tumour is quite small in relation to body weight, leads to rectification of anaemia before measures to control the tumour are undertaken.

Contrasting with the clinical attentiveness to constitutional depradations associated with localised cancer is the frequent neglect of such considerations in the experimental study of rodent tumours. In our experience, rapidly increasing host anaemia is frequently present when transplanted mouse tumours attain a size at which significant observations of the tumour are likely to be made. For example, terminal depression of tumour growth rate (Laird, 1964) and the micro-anatomy of extremely large tumours (Inch and McCredie, 1968) have been interpreted without reference to the possible implication of progressive host anaemia in the changes described.

Since the oxygenation of tumours has a potent influence on their response to irradiation, anaemia of the host deserves consideration in the interpretation of both clinical and experimental radiotherapy findings.

Comparisons of the responses to therapy of anaemic and non-anaemic clinical cases of cancer (*e.g.* Evans and Bergsjø, 1965) are complicated by the strong possibility that anaemic cases are more likely than non-anaemic cases to harbour malignancies which are more advanced or more aggressive. Although such inadvertent selection can be avoided in experimental investigation of the effect of anaemia on response to therapy, very few such studies have in fact been made.

In the present paper we describe experiments in which we have studied the influence of chemically-induced anaemia on the viability and radiosensitivity of murine tumour cell populations present as visceral infiltrations or as solid tumours. Differences in the response of these two types of population are of interest in relation to problems associated with the presence of hypoxic cells in solid tumours subjected to radiotherapy.

### MATERIALS AND METHODS

### *Mice and tumours*

Mice of strains CBA/Ht or WHT/Ht were used in all the experiments; these were bred in this Unit by sib-mating and were aged 2–4 months at the time of experiment. All the transplanted tumours used arose in and were maintained in the same colony of isologous mice.

*CBA leukaemia "Th"* is a lymphoblastic strain which grows as an ascites tumour after intraperitoneal injection but which produces ascites-free infiltration of the viscera after injection by other routes. A single cell of this strain will effect transplantation. Material used in the present experiments was from the 442nd to 525th serial passages.

*CBA leukaemia R–I* 1 is a radiation-induced lymphocytic leukaemia which produces ascites-free infiltration of the viscera after injection by any route. Material used was from passages 201–208.

*WHT ascites tumour* 1 is of spontaneous origin and was in its 131st serial passage when used in the experiment to be described.

*CBA sarcoma F* is an anaplastic tumour which grows as discrete solid tumours after subcutaneous injection and has been used in several previously reported studies (Hewitt, 1966; Hewitt and Blake, 1968; Baker, Lindop and Hewitt, 1968). The present experiments were done with material from the serial passage range 365–377.

*WHT squamous carcinoma* is a differentiating tumour of spontaneous origin whose characteristics have been described previously in detail (Hewitt, Chan and Blake, 1967). Experiments to be described were done with material from serial passage No. 97 and 135.

### *Treatment with phenylhydrazine hydrochloride (PH)*

The required dose of the drug was always given in 0·2 ml. of phosphate-buffered saline. In the experiment using WHT ascites tumour 1, the drug was given by subcutaneous injection to avoid direct contact between drug and tumour cells; in all other experiments the drug was given intraperitoneally.

*Measurement of the haemoglobin (Hb) level in the peripheral blood*

Blood samples were obtained from the tail after guillotine amputation of the tip, or from the exposed auricles of anaesthetised mice when further survival was not required. A volume of 20 $mm^3$ of blood was diluted in 4 ml. of distilled water to effect haemolysis, and centrifuged to remove the cloudiness which appears in solutions of blood from PH-treated mice. The optical density of the supernatant fluid was measured at 475, 510 and 576 nm. The optical density at 475 nm. is a measure of the total concentration of Hb, including both oxy- and met-Hb. The factor (OD at 576 nm.)/(OD at 510 nm.) is lineally related to the fraction of Hb present as met-Hb (Wintrobe, 1951), and the latter value was obtained from a graph of the relation. The level of oxy-Hb in the blood was calculated from the total Hb and the size of the met-Hb fraction, and was expressed as a percentage of the mean value for normal mice of the strain used. The Hb value of tail blood was usually 8 per cent higher than that of heart blood. Very exceptionally, a small fraction of met-Hb was found in the blood of untreated mice.

*Transfusion of erythrocytes*

Blood for transfusion was collected by heart puncture from heparinised mice of the same strain as the intended recipients. The erythrocytes were washed twice in phosphate-buffered saline and stored as packed cells at 4° C. until used. Volumes of 0·3–0·8 ml. of packed cells were transfused usually by the intravenous route and very occasionally by the intraperitoneal route. In experiments in which transfused mice were subsequently irradiated, the two procedures have been separated by at least 30 minutes to allow at least some recovery from the early haemodynamic disturbance to be expected from large volume transfusion.

*Exposure to oxygen under high pressure (OHP)*

Mice to be irradiated in OHP were placed in a pressurisation vessel described previously (Hewitt, 1966). The oxygen pressure was raised to 3 atmospheres absolute pressure at a rate of 10 p.s.i./min. Irradiation was delivered after equilibration of the mice for 15 minutes at this pressure. A gas flow rate of 3 l./min. was maintained throughout.

*Irradiation*

Individual mice were exposed to single-doses of whole-body irradiation using a therapy machine operated at 250 kV and 15 mA with filtration of the rays through 0·5 mm. Cu and 1·0 mm. Al. The exposure dose rate was always 156 R/min. Mice were exposed in air or OHP, or were killed by neck fracture immediately before irradiation when anoxic conditions were required.

*Measurement of cell survival among clonogenic tumour cells irradiated* in vivo *or in killed mice*

The methods used to prepare single-cell suspensions from infiltrated viscera or solid tumours have been described previously (Hewitt, 1958, 1966; Hewitt *et al.*, 1967). These publications also describe the technique of quantitative transplantation used to measure the proportion of clonogenic cells in counted cell suspensions prepared from unirradiated and irradiated tissues, and the means of deriving cell surviving fractions from such data.

Technical details relating to particular experiments are described in the following section.

### EXPERIMENTS AND RESULTS

### *Effects of phenylhydrazine Hydrochloride (PH) on Normal Mice*

Mice receiving 180 mg./kg. of the drug showed a mortality of 5 per cent, all deaths occurring within 24 hours. Death was preceded by signs of " air hunger " and convulsions. Figure 1 shows the changes in haemoglobin level in WHT mice at intervals after a single intraperitoneal dose of 180 mg./kg. There is a rapid fall to about 30 per cent, and this minimum value is sustained for the first 3 days; thereafter there is rapid recovery, to over 80 per cent by the 9th day The percentage of total haemoglobin present as methaemoglobin remains at about 25 for the first 3 days, falling to zero between the 4th and 5th days. Mice which received an initial dose of 180 mg./kg. followed by daily maintenance doses of 36 mg./kg. from the 4th to 13th day after the initial dose showed slower recovery. During the later stages of recovery, almost all the circulating erythrocytes were reticulocytes and there was some leucocytosis.

Since it is proposed to interpret the results of the experiments to be described in terms of tissue hypoxia, an assurance is required that the effects of the drug are confined to the erythrocytes and do not include direct cytotoxic effects on proliferating cell populations. The following observations on mice receiving high doses are, indeed, characteristic of severe hypoxaemia: the onset of convulsions and " air hunger " can be prevented or delayed by placing the mice in pure

FIG. 1.—Recovery of haemoglobin levels in WHT mice after a single dose of 180 mg./kg. of phenylhydrazine hydrochloride. Each point was contributed by values from three mice oxygen; in mice surviving high doses we have observed azotaemia and necrosis of the tip of the tail and of the edge of the ventral lobe of the liver. The absence of damage to proliferating cell populations is shown by the following observations: there is no diarrhoea; the rate of recovery from PH-induced anaemia is similar to that from haemorrhagic anaemia; there is no sustained leucopoenia; and transplantation assays of malignant cells from PH-treated mice revealed no evidence that morphologically intact tumour cells have received damage to their reproductive integrity.

### *Effect of PH on the Size of Malignant Cell Populations* in vivo

1. *Ascites tumour*

Three groups each of 12 WHT female mice received equal intraperitoneal inocula of cells of WHT ascites tumour 1. On the 7th day after injection the mice of one group were killed and the following procedure was carried out on each mouse: the volume of ascitic fluid was determined by weighing the mouse before and after the complete removal of fluid; an aliquot of the fluid was diluted in heparinised buffered saline and the cell density in the undiluted fluid was determined by counting the cells in a haemocytometer; the total number of ascites cells was calculated from the values for volume and cell density. At the time of examination of the first group of mice, all mice of the second group received a subcutaneous injection of 4 mg. of PH. Forty-eight hours after injection, haemoglobin levels were determined in the treated group and in the third (untreated) group. Ascitic fluid volumes and cell densities were determined as for the first control group.

TABLE I.—*Ascites Tumours*

| Group | Day | Body weight (g.) | Tumour volume (ml.) | Cell density per ml. ($\times 10^{-8}$) | Total cells per mouse ($\times 10^{-8}$) | Hb% |
|---|---|---|---|---|---|---|
| Control | 7 | $26\cdot3\pm0\cdot9$ | $3\cdot1\pm1\cdot5$ | $1\cdot84\pm0\cdot52$ | $5\cdot05\pm1\cdot71$ | — |
| Control | 9 | $25\cdot8\pm2\cdot7$ | $9\cdot4\pm1\cdot7$ | $0\cdot58\pm0\cdot07$ | $5\cdot36\pm1\cdot13$ | $77\pm4$ |
| PH-treated | 9 | $24\cdot7\pm2\cdot0$ | $2\cdot05\pm0\cdot75$ | $0\cdot77\pm0\cdot51$ | $1\cdot30\pm0\cdot82$ | $17\pm4$ |

Values are: means $\pm$ S.D.

Table I shows the results of these examinations. Between the 7th and 9th days after transplantation of ascites cells the untreated control mice showed no significant increase of total cell content but exhibited a substantial increase in ascitic fluid volume and reduction of cell density. Assuming that the values for mean fluid volume and cell density for the control group on the 7th day after transplantation are representative of the mean values for the PH-treated mice at the time of treatment, it is seen that the effect of the drug was to reduce both the fluid volume and the cell density; the mean total number of cells per mouse was reduced to almost one quarter during the 48 hours following treatment; the considerable rise in fluid volume seen in the untreated mice between the 7th and 9th days after transplantation was prevented by treatment with PH. It is seen that, although the treated mice sustained very severe anaemia, the loss of body weight in the subsequent 48 hours was not significantly greater than that in the untreated mice.

Our conclusion from this experiment is that induction of severe anaemia in the ascitic mice was associated with a significant loss of ascites cells and that this loss was due to relative deprivation of available oxygen. It is of interest that the cells which were lost evidently underwent complete disintegration and absorption within the 2-day period of anaemia.

2. *Leukaemic livers*

The development of generalised leukaemia in mice to which isologous leukaemia cells have been intravenously transplanted is associated with progressive increase of liver weight without the formation of ascites tumour; in the terminal stages of the disease, using CBA leukaemia R–I 1, the liver may attain over four times its normal weight. Histological examination shows that the increment of weight can be ascribed to masses of leukaemia cells occupying the sinusoids and permeating the portal tracts. Measurement of the radiosensitivity of leukaemia cells in such heavily infiltrated livers reveals that the proportion of leukaemia cells which are severely hypoxic and relatively radioresistant in mice breathing air remains below 0·1 per cent. The absence of appreciable severely hypoxic foci must be attributed to the large vascular reserve of the liver. It was of interest to study the effect of severe anaemia on the size of the infiltrating leukaemia cell population.

Sixty female CBA mice were injected intravenously with 9000 cells of CBA leukaemia R–I 1. At intervals after transplantation individual mice or small groups were killed by bleeding from the severed carotid arteries under ether anaesthesia. All lobes of the liver were excised and the total liver weighed. Between the 9th and 14th days after transplantation, there was a progressive rise in total liver weight from the normal weight of 1 g. to a mean weight of 2·9 g. On the 14th day, each of a group of 19 leukaemic mice received 125 mg./kg. of PH intraperitoneally; thirty hours later, by which time 12 of the treated mice had died, liver weights were determined for the 7 PH-treated survivors and for 10 untreated leukaemic mice. In Fig. 2, individual liver weights are recorded graphically in relation to the time after transplantation; there is a wide variation of values within the later groups, but comparison of the values for control mice examined on the day of injection of PH with those for PH-treated mice examined 30 hours later shows that treatment with PH was associated with a dramatic loss of liver weight in at least some of the mice. Similar but less striking results were obtained in a similar experiment using a different strain of leukaemia. In a subsequent experiment, 12 normal female CBA mice received a single dose of 125 mg./kg. of PH and the liver weights were determined 27 hours later. The mean liver weight was 1·08 g., compared with 1·06 g. in untreated controls.

The haemoglobin values of leukaemic mice on the day of treatment with PH were about 95 per cent. The high mortality in the treated mice is not, therefore, due to the superimposition of induced on existing anaemia, and may possibly be due to the absorption of a very large mass of killed cells, the débris of which would have direct access to the circulation.

### *Effect of Anaemia on the Radiosensitivity of Leukaemia Cells in the Livers of Leukaemic Mice*

Figure 3 shows the radiosensitivity of leukaemia cells of strain CBA " Th " irradiated either *in vivo* in mice breathing air (left hand curve) or under anoxic conditions (in recently killed mice). The survival curves drawn are described

FIG. 2.—Total liver weights of CBA mice at intervals after transplantation of leukaemia cells. ○, untreated mice; ●, mice receiving a single dose of phenylhydrazine 30 hours previously. Points are individual values; bars are mean values.

FIG. 3.—Survival of leukaemia cells in the livers of CBA mice after irradiation: ○, in air; ◑, in air after treatment with phenylhydrazine; ●, after killing.

by Do values of 120 R and 280 R, and the oxygen enhancement ratio is 2·3. These values are based not only on the present data but on previous data for the same strain of leukaemia. Also shown in Fig. 3 are survival points for cells irradiated *in vivo* in mice which had received 120–140 mg./kg. of PH within 24 hours before irradiation. The mice had Hb levels of between 21 and 43 per cent (mean 34 per cent) at the time of irradiation. If the survival points for irradiation of anaemic mice are assumed to lie on the anoxic segments of compound curves describing the radiosensitivity of a mixed population of oxic and hypoxic cells, the percentage of hypoxic cells which were anoxic lay between 10 and 100 per cent (mean 34 per cent). Thus, a substantial proportion of the leukaemia cells in the livers had changed from an oxic to an hypoxic status following the induction of anaemia. Similar findings were obtained using CBA leukaemia R–I 1.

*Effect of transfusion of Blood on the Radiosensitivity of Leukaemia Cells in Anaemic Mice*

Six pairs of mice with moderately advanced CBA leukaemia " Th " were rendered severely anaemic by the injection of PH within 24 hours of exposure to irradiation in air. Between 30 minutes and 3 hours before exposure, one mouse of a pair received a transfusion of 0·5–0·8 ml. of packed CBA erythrocytes. Each pair was exposed to whole-body irradiation, after which the level of haemoglobin was measured and leukaemia cells were released from the minced livers and assayed for determination of the cell surviving fraction. The results of the paired experiments are recorded in Table II. The percentage of leukaemia cells which

TABLE II.—*Effect of Transfusion on Radiosensitivity of Leukaemia cells in PH-treated Mice*

| Transfusion | Hb % during irradiation | Dose X-rays (R) | Liver weight (g.) | Surviving fraction ($\log_{10}$) | % Cells anoxic |
|---|---|---|---|---|---|
| − | 43 | 1200 | 1·7 | $\bar{2}\cdot30$ | 71 |
| + | 85 | 1200 | 1·3 | $\bar{3}\cdot23$ | 6 |
| − | 21 | 1300 | 1·2 | $\bar{2}\cdot39$ | 100 |
| + | 102 | 1300 | 1·5 | $\bar{4}\cdot78$ | 3 |
| − | 35 | 1400 | 1·3 | $\bar{3}\cdot44$ | 20 |
| + | 87 | 1400 | 1·6 | $\bar{4}\cdot76$ | 4 |
| − | 39 | 1500 | 1·7 | $\bar{3}\cdot65$ | 45 |
| + | 66 | 1500 | 1·5 | $\bar{4}\cdot24$ | 2 |
| − | 35 | 1500 | 1·4 | $\bar{4}\cdot95$ | 9 |
| + | 74 | 1500 | 1·4 | $\bar{4}\cdot19$ | 2 |
| − | 29 | 1600 | 1·2 | $\bar{4}\cdot88$ | 11 |
| + | 98 | 1600 | 1·2 | $\bar{5}\cdot66$ | 0·7 |

were hypoxic during irradiation has been determined from the vertical depression of the survival points below the curve for purely hypoxic cells shown in Fig. 3. The percentage of anoxic cells in untransfused mice, which varies quite widely, is not correlated either with liver weight or with haemoglobin level at the time of irradiation. However, in all cases the percentage of hypoxic cells in a transfused mouse is substantially lower than in the untransfused mouse of the same pair; the mean factor difference under the two conditions varies between 4 and 33 (mean 16). Thus, the hypoxic status of a high proportion of the leukaemic cells present in the livers of anaemic mice can be raised to an oxic status by transfusion of blood. Nevertheless, all the survival points for cells from transfused mice lay at least one decade above the curve for fully oxygenated cells shown in Fig. 3, so that transfusion did not succeed in reducing the proportion of hypoxic resistant cells to the very low levels found in non-anaemic leukaemic mice.

### *Effect of OHP-Breathing on the Proportion of Leukaemia Cells which are Hypoxic in Severely Anaemic Mice*

Three pairs of CBA mice with moderately advanced CBA leukaemia " Th " received a single dose of 120–150 mg./kg. of PH. Twenty-four hours after the administration both mice of a pair were exposed to the same dose of irradiation, one being exposed in air, the other in OHP. After irradiation, the haemoglobin level of each mouse was measured and leukaemia cells from the liver were assayed for determination of the cell surviving fraction. The results of the three paired experiments are recorded in Table III and Fig. 4. In mice breathing air the percentages of cells which were anoxic, by the same criteria as were used in the previous experiment, lay between 6 and 10. The results of the assays of cells from mice breathing OHP could only be expressed as maximum values because

TABLE III.—*Effect of OHP-breathing on Radiosensitivity of Leukaemia Cells in Anaemic Mice*

| Gas breathed | Dose X-rays (R) | Liver weight (g.) | Hb % | Surviving fraction ($\log_{10}$) | % Cells anoxic |
|---|---|---|---|---|---|
| air | 1400 | 1·2 | 28 | $\bar{4}\cdot85$ | 6 |
| OHP | 1400 | 1·1 | 28 | $\leqq\bar{5}\cdot08$ | $\leqq0\cdot08$ |
| air | 1800 | 1·3 | 38 | $\bar{4}\cdot42$ | 8 |
| OHP | 1800 | 1·2 | 31 | $\leqq\bar{6}\cdot46$ | $\leqq0\cdot08$ |
| air | 1900 | 1·1 | 26 | $\bar{4}\cdot39$ | 10 |
| OHP | 1900 | 1·2 | 26 | $\leqq\bar{7}\cdot67$ | $\leqq0\cdot02$ |

FIG. 4.—Survival of leukaemia cells in the livers of anaemic CBA mice after irradiation *in vivo*: ◑ in air; ○, in OHP. The curves drawn have been superimposed from Fig. 3.

the actual TD50 values were above the range of cell numbers assayed. However, it is quite evident that the increased oxygen carried in the blood during breathing of OHP was sufficient to oxygenate practically all the severely hypoxic cells present in the livers of the anaemic mice. A comparison of Tables II and III does, indeed, suggest that oxygenation was more effective during OHP-breathing than after transfusion. Unfortunately, the two sets of experiments were done at different times and their results are not strictly comparable. Nevertheless, we shall refer later to theoretical considerations justifying a possible superiority of OHP-breathing over transfusion.

### *Effect of Prolonged Anaemia Followed by Transfusion on the Radiosensitivity of Sarcoma Cells* in vivo

CBA sarcoma F was used in the present experiments. Previous reports have indicated that this tumour contains an exceptionally high proportion of severely hypoxic cells in mice breathing air (Hewitt and Wilson, 1961), and that this proportion is not significantly reduced in mice breathing OHP (Hewitt, 1966). In the present experiments we allowed subcutaneously transplanted tumours to grow in hosts which were maintained in a severely anaemic state by the repeated injection of PH. One of a pair of such anaemic mice was transfused with packed cells before exposure of both mice to whole-body irradiation. After measurement of the Hb levels of the irradiated mice, a single-cell suspension was prepared from the two tumours of each mouse and the cell surviving fraction was measured by transplantation assay. Cell survival was compared in the tumours from the transfused and untransfused mouse.

The rationale of the experiment was as follows: the length of oxygen gradients extending from capillaries into viable tumour tissue would be directly related to the concentration of oxygen in the blood (Thomlinson and Gray, 1955); in tumours grown up in severely anaemic hosts, the length of O gradients into viable tumour tissue would be shorter than in non-anaemic mice, and the proportion of vascular to viable tumour tissue should be greater; when the concentration of oxygen carried in the blood is restored to normal levels by transfusion, we expect the length of $O_2$ gradients from vessels to be increased and to extend into regions of the tumour which had been previously hypoxic; such an effect would be registered by a lower survival of clonogenic cells in irradiated tumours of transfused mice than of untransfused mice.

Approximately 30,000 sarcoma F cells were injected subcutaneously into each axilla of the mice. On the 5th day after transplantation, a loading dose of PH was administered to the mice; suitable maintenance doses of PH were given on subsequent days to keep the Hb level in the range 25–45 per cent until the day of irradiation. One of a pair of equally treated mice was transfused with packed erythrocytes before exposure to irradiation; the interval between transfusion and irradiation was varied between 2·5 and 20 hours in different experiments. After exposure to irradiation, the Hb levels of the mice were determined and the tumours were excised and weighed; cell suspensions prepared from the tumours were assayed for determination of the TD50, and the surviving fraction was calculated using as the control value the mean TD50 for unirradiated sarcoma cells assayed in the presence of an excess of radiation-killed cells.

The results of six paired experiments, recorded in Table IV, showed only a very slight effect of transfusion on cell survival. Although the surviving fraction

TABLE IV.—*Effect of Transfusion on Radiosensitivity of Sarcoma Cells in PH-treated Mice*

| Transfusion | Hb % during irradiation | Dose X-rays (R) | Mean tumour weight (g.) | Surviving fraction ($\log_{10}$) |
|---|---|---|---|---|
| − | 41 | 1900 | 0·7 | $\bar{3}$·23 |
| + | 100 | 1900 | 1·2 | $\bar{3}$·04 |
| − | 35 | 2100 | 1·3 | $\bar{3}$·00 |
| + | 80 | 2100 | 0·8 | $\bar{4}$·68 |
| − | 33 | 2100 | 1·0 | $\bar{4}$·90 |
| + | 63 | 2100 | 1·3 | $\bar{4}$·76 |
| − | 24 | 2200 | 0·5 | $\bar{4}$·55 |
| + | 95 | 2200 | 0·5 | $\bar{4}$·77 |
| − | 45 | 2300 | 1·2 | $\bar{4}$·63 |
| + | 82 | 2300 | 0·8 | $\bar{4}$·15 |
| − | 49 | 2400 | 0·6 | $\bar{4}$·56 |
| + | 114 | 2400 | 0·7 | $\bar{4}$·40 |

was smaller in the transfused mouse in 5/6 pairs, the mean value of SF transfused/SF untransfused was 0·75. An observation of interest was that the gross appearance of tumours from PH-treated anaemic mice suggested that they were well vascularised and free from massive necrosis. During excision of tumours from transfused mice a considerable amount of vascular congestion was observed in the peritumoral tissue.

### *Serial Treatment of Solid Tumours by Irradiation and Subjection to Systemic Insults*

It is reasonable to assume that the hypoxic cells present in all solid tumours that have been suitably examined, being evidently located in relatively poorly vascularised situations, would be least able to withstand devaluation of blood-borne nutrients, including oxygen. On the other hand, well-oxygenated cells would be relatively more sensitive to lethal damage by irradiation. Following this consideration, we have done numerous experiments in which irradiation has been preceded or succeeded by subjection of the host to a period of severe anaemia, immersion in hypobaric (7 per cent) oxygen for over 12 hours, insulin-induced hypoglycaemia, or acidosis induced by the injection of lactic acid, or by a combination of these treatments. When irradiation was given after the exposure to systemic insult, the constitutional depredations were restored before irradiation. Tumour response has been compared in equally irradiated mice which did or did not receive additional treatment. Response was measured in terms of the fractional survival of clonogenic cells or of the rate and extent of regression of tumour volume. In none of these experiments has a significant influence of the systemic insults been apparent. For example, Fig. 5 shows survival points for cells of the WHT squamous carcinoma irradiated as subcutaneous tumours. One mouse received irradiation only, one sustained severe anaemia, hypoglycaemia and acidosis for 48 hours before transfusion and irradiation, and one sustained anaemia and hypoglycaemia during the 24 hours preceding transfusion and irradiation. The cell survival points for cells from the three tumours all lie close to the curve defined previously for this tumour irradiated in mice breathing air (Hewitt *et al.*, 1967). The relationship between the curve for purely hypoxic cells and that for cells from tumours irradiated in air indicates that 18 per cent of the cells were hypoxic under these conditions.

FIG. 5.—Survival of cells of WHT Squamous Carcinoma irradiated in air as solid tumours *in vivo*. ○, irradiation only; ●, sequential treatment with irradiation and multiple systemic insults, as described in text. The continuous and interrupted lines for tumours irradiated under anoxic conditions or in air respectively have been superimposed from previous data (Hewitt *et al.*, 1967).

### DISCUSSION

In interpreting the experiments described it has been assumed that PH does not directly damage proliferating cells. The assumption is justified not only by safe usage of the drug in clinical medicine over several decades for the treatment of polycythaemia vera, but by a variety of observations recorded in this paper and elsewhere. The rate of recovery from severe anaemia induced by the drug, as shown in Fig. 1, was similar to that from purely haemorrhagic anaemia; the reproductive integrity of malignant cells from mice which had received high doses was unimpaired; and no sign attributable to damage to the intestinal mucosa or leucocytic systems was observed. Grasso and Shepherd (1968) have shown that haemopoietic recovery occurred in newts which had received single doses of PH sufficient to destroy all the circulating erythrocytes. Smith (1970) demonstrated a rise in the resistance of mice to whole-body irradiation given 7 days after large doses of the drug; he attributed this resistance to an increase in the haemopoietic stem cell population. From all this evidence, it appears that a " chemotherapeutic " effect of the drug can be excluded and that the results of our experiments can be confidently attributed exclusively to reduction of tissue oxygenation associated with the drug-induced anaemia. However, it would be unwise to attribute these deficiencies of tissue oxygenation directly to reduction of the oxygen-carrying capacity of the blood; the anaemia induced was severe enough to give rise to cardiovascular insufficiency which could lead to changes in the rate of flow of blood through tissues; additionally, we found evidence of hydraemia, urinary suppression and azotaemia in severely anaemic mice.

Our demonstration of fairly rapid loss of malignant cells from ascites tumours and leukaemic livers suggests that malignant cells of this type may undergo fairly rapid plasmolysis under certain conditions of oxygen deprivation. It is possible that the " cell loss factor " revealed by kinetic studies of solid tumours (Steel, 1968) is associated with the continual passage of cells into hypoxic regions of the tumour.

In a previous publication (Hewitt, 1967) it was shown that the leukaemia cell population in the livers of leukaemic mice increases exponentially and that the proportion of cells which are hypoxic remains at a very low level even in livers which are heavily infiltrated. The effect of inducing severe acute anaemia in the hosts bearing such infiltrated livers is evidently to cause death and dissolution of a proportion of the cells, to leave others viable but hypoxic, and to leave others relatively well-oxygenated; our data does not allow us to state what proportion of the cells are of intermediate oxygenation and radiosensitivity.

A comparison of Tables II and III suggests that OHP-breathing is more effective than transfusion in reducing the proportion of hypoxic cells in the livers of leukaemic mice, although transfusion would effect the greater degree of relative hyperoxaemia. A possible explanation of this unexpected finding is that the large volume transfusions may have produced haemodynamic disturbance detrimental to blood flow and possibly damaging to blood vessels, whereas OHP-breathing would be free from such disturbance.

Our experiments with the solid tumours were instigated by our understanding of the rationale for use of OHP-breathing during radiotherapy, as originally proposed by Gray, Conger, Ebert, Hornsey and Scott (1953). This is, that the anoxic cells in solid tumours are situated at the extremity of oxygen gradients and can be oxygenated by extension of such gradients. This hypothesis was well substantiated by our studies of hypoxic leukaemia cells in the livers of anaemic mice. However, our failure to demonstrate a significant reduction of hypoxic cells in the sarcomas by the procedures described, combined with our previous failure to demonstrate a significant effect of OHP-breathing on the proportion of hypoxic cells in the same tumour (Hewitt, 1966), seems to us to invite caution in accepting a hypothesis that a majority of the hypoxic cells, at least in this tumour, are critically situated along oxygen gradients.

Hill, Bush and Yeung (1971), measuring cell survival fractions after single-dose irradiation of transplanted C3H sarcomas, demonstrated that 12 per cent were hypoxic when the hosts had Hb levels of about 9·5 g per cent, the anaemia being due to the systemic effects of the growing tumour. The hypoxic cells fell to 6 per cent when the hosts were transfused up to normal Hb levels before irradiation. The findings of these authors and those reported here suggest that at least half of the hypoxic cells fail to become oxygenated following transfusion. The question arises, whether these residual hypoxic cells are so far removed from nutrient sources that they would have succumbed to inanition had they not been " rescued " from the tumour and retransplanted as required by the assay procedure. Certainly, a very significant proportion of these relatively inaccessible cells are not so doomed. Suit and Maeda (1967) showed that the single dose of irradiation in OHP required to control 50 per cent of mouse mammary tumours of 250 $mm^3$ is consistent with 2–7 per cent of the cells in these tumours remaining hypoxic and viable and contributing to a recurrence of growth.

It is reasonable to ascribe the hypoxic status of a proportion of the cells in solid tumours to their location within the vascular framework of the tumour. Indeed, the morphological observations of Thomlinson and Gray (1955), Reinhold (1967) and Tannock (1968) leave no doubt of the importance of such topographical considerations. Nevertheless, it is certain that functional differences relating to vascular efficiency are super-imposed on any microanatomical pattern observed and that the tumour is prone to vascular accidents resulting in infarcts of variable extent. Our present inability to measure the effect of such influences in the way required inevitably limits interpretation of the experiments we have described.

We are grateful to Miss Angela Walder for the breeding and care of all the mice used and for other technical assistance. The cost of the research was met exclusively by the Cancer Research Campaign.

### REFERENCES

Baker, D. J., Lindop, P. J. and Hewitt, H. B.—(1968) *Br. J. Radiol.*, **41**, 318.
Evans, J. C. and Bergsjø, P.—(1965) *Radiology*, **84**, 709.
Grasso, J. A. and Shepherd, D. C.—(1968) *Nature, Lond.*, **218**, 1274.
Gray, L. H., Conger, A. O., Ebert, M., Hornsey, S. and Scott, O. C. A.—(1953) *Br. J. Radiol.*, **26**, 638.
Greenfield, R. E., Godfrey, J. E. and Price, V. E.—(1958) *J. natn. Cancer Inst.*, **21**, 641.
Hewitt, H. B.—(1958) *Br. J. Cancer*, **12**, 378.—(1966) *Br. J. Radiol.*, **39**, 19.—(1967) *Proc. int. Conf. Radiat. Biol. and Cancer*, Kyoto, Japan, p. 9.
Hewitt, H. B. and Blake, E. R.—(1968) *Br. J. Cancer*, **22**, 808.
Hewitt, H. B., Chan, D. P. and Blake, E. R.—(1967) *Int. J. Radiat. Biol.*, **12**, 535.
Hewitt, H. B. and Wilson, C. W.—(1961) *Ann. N.Y. Acad. Sci.*, **95**, 818.
Hill, R. P., Bush, R. S. and Yeung, P.—(1971) *Br. J. Radiol.*, **44**, 299.
Inch, W. R. and McCredie, J. A.—(1968) *Can. med. Ass. J.*, **99**, 337.
Laird, A.K.—(1964) *Br. J. Cancer*, **18**, 490.
Reinhold, H. S.—(1967) " Stralingsgevoeligheid van Tumoren ". Thesis. Rotterdam.
Smith, L. H.—(1970) *Trans. N.Y. Acad. Sci.*, **32**, 448.
Steel, G. G.—(1968) *Cell Tissue Kinetics*, **1**, 193.
Suit, H. D. and Maeda, M.—(1967) *J. natn. Cancer Inst.*, **39**, 639.
Tannock, I. F. —(1968) *Br. J. Cancer*, **22**, 258.
Thomlinson, R. H. and Gray, L. H. —(1955) *Br. J. Cancer*, **9**, 539.
Wintrobe, M. M.—(1951) in " Clinical Hematology ", 3rd edition. London (Henry Kimpton) p. 325.